# Prevalence of visual impairment in relation to the number of ophthalmologists in a given area: a nationwide approach

**DOI:** 10.1186/1477-7525-4-34

**Published:** 2006-06-06

**Authors:** Antoine J Lafuma, Antoine P Brézin, Francis L Fagnani, Mounir Mesbah, Gilles H Berdeaux

**Affiliations:** 1 Cemka, 43, Boulevard du Maréchal Joffre, F-92340 Bourg-la-Reine, France; 2 Centre Hospitalier Universitaire Cochin, Service d'Ophtalmologie, 24 rue du Faubourg Saint Jacques, F-75014 Paris, France; 3 Université Pierre Et Marie Curie, (UPMC), Laboratoire de Statistique Theorique et Appliquée,175 rue du Chevaleret, F-75013 Paris, France; 4 Conservatoire National des Arts et Métiers, 292, rue Saint-Martin, F-75003 Paris, France; 5 Alcon France. 4 Rue Henri Sainte Claire Deville, F-92563 Rueil-Malmaison, France

## Abstract

**Background:**

Sociological and economic risk factors of visual impairment have never been described in France at a national level as the association between the number of ophthalmologists per inhabitant and visual impairment prevalence.

**Methods:**

Two national surveys were pooled. First, 2075 institutions were selected at random from the French Health Ministry files. Second, a random, stratified sample of 356,208 citizens living in the community was selected. Blindness and low vision (LV) prevalence rates were estimated by age and gender. Geographical equities were estimated by logistic regression adjusted on age and occupational category. The association between ophthalmologist density and visual impairment prevalence rate was estimated *per *region. Interviews were completed with 14,603 (94.9%) of 15,403 randomly selected subjects in institutions, and 16,945 (77.8%) of 21,760 randomly selected subjects in the community. Three groups were defined from the interviews: low vision, blind, and control.

**Results:**

Prevalence rates were LV 2.08% and blindness 0.12%. Both rates increased exponentially with age. No major difference was found with gender. Injury was the declared reason for both LV (12%) and blindness (12%). Large regional differences in prevalence persisted for LV after adjustment on age and occupation (ORs: 0.35 to 2.10), but not for blindness. Regions with ophthalmologists below the national *per capita *average were usually those with higher LV prevalence.

**Conclusion:**

An inverse correlation was found between ophthalmologist number and LV prevalence rates for subjects of similar age and socio-professional category. This denoted possible inequity in the provision of healthcare.

## Background

Visual impairment was responsible for 2,286,000 'disability adjusted life years' in the high income countries in 2001 [1]. The cost of blindness to the Australian government and community was estimated at between AUS$ 9,749 and AUS$ 26,720 per patient per year [2]. The mean cost per blind person per year was US$ 11,896 in 1990 in the USA and totalled US$ 4 billion [3]. Therefore, it is crucial to obtain nationwide estimates of low vision and blindness prevalence to allocate the right amount of resources especially when life expectancy is predicted to increase [4,5]. It is also important to understand the causes of visual impairment, in order to implement adequate preventative activities.

According to the International Statistical Classification of Diseases, Injuries and Causes of Death, visual impairment includes both low vision and blindness. Low vision is defined as visual acuity less than 6/18, but equal to or better than 3/60, or a corresponding visual field loss to less than 20 degrees in the better eye using best possible correction. Blindness is defined as visual acuity less than 3/60, or a corresponding visual field loss to less than 10 degrees in the better eye using best possible correction [6].

Primary open-angle glaucoma and age-related macular degeneration are the main two diseases leading to blindness in Western developed countries. Apart from cataract surgery, treatments are available which at best maintain vision, or otherwise postpone visual acuity deterioration. A significant portion of the burden caused by visual impairment is borne by families and includes rehabilitation, medical devices, dedicated software, home modifications, caring time, loss of family revenue, etc.. Nationwide extrapolation has shown that the non-medical costs of visual impairment were comparable to the nationally reimbursed drug budget [7]. It is therefore crucial to obtain nationwide estimates of low vision and blindness prevalence rates so that sufficient resources are allocated appropriately (medical and non-medical), especially when increasing life expectancy is predicted to continue [8].

The use of registers to estimate the prevalence of blindness is controversial, since a high proportion of visually impaired subjects do not register [9-12]. According to a WHO review on the prevalence of blindness, ten surveys were conducted in Europe up to 1994 [13]. An update was performed in 2002 [14]. Most studies were conducted at a local level, using direct standardisation to derive national estimates. This technique was used by the Eye Diseases Prevalence Research Group [15]. However, local surveys do not estimate disparities in prevalence rate amongst different geographical areas.

Healthcare expenditure has increased substantially in all Western industrialised countries during the last decades [16]. As a result, efficiency in resource allocation has become a major issue in public health decisions, but equity is very important, too, as stated by the National Institute for Clinical Excellence [17]. Equity is necessary to ensure that two patients, suffering from a similar disease, have access to the same quality of care, and experience the same clinical outcome. However, equity and efficiency (cost per unit of production) are incompatible [18], so political decisions must be made. Such decisions should be based on studies aimed at quantifying acceptable levels of *in*equity, in order to accommodate fixed budgets. Little has been published on equity and eye care delivery [19].

The issue of equity might differ according to healthcare systems, e.g., as between France and the United Kingdom. Some econometric surveys confirm the existence of 'physician-induced demand' in the French system of ambulatory care, which causes healthcare expenditure to increase [20]. This relationship has been used for decades to justify limits on the number of students entering medical schools. It is contrary to the idealistic theory that an optimal number of physicians would maximise efficient healthcare provision. In this context, a link between the demography of ophthalmologists and the prevalence of both low vision and blindness has never been studied.

The present survey had three aims: (1) to identify patient demographic risk factors of visual impairment; (2) to compare a visual impairment index across the different regions of France; and (3) to study the relationship between this index and ophthalmologist demographics.

## Methods

Data were gathered in two surveys by the Institut National de la Statistique et des Etudes Economiques (INSEE) [21]. The databases were subsequently made available to researchers for secondary analyses. The methodology of these two surveys has already been described [22-24] elsewhere. The following is a condensed description which should help readers to understand and interpret the results.

### Experimental design: the community survey

A national census survey is performed every ten years in France. Each household is visited by an interviewer and data are collected on each member of the family. Information was provided by one person of the household. All French people (no age limits) are questioned and answering is compulsory.

A "Handicap-Dependency" survey documented "handicap", incapacity and dependency of French citizens, living in the community, at a national level. It was decided to piggy-back on the 1999 national census survey.

The survey followed guidelines and principles for developing disability statistics, published by the United Nations [25]. The sample was selected by a two-step process [26,27].

1. In 1990, 57,831,816 citizens were documented and statistics on geographical area were available. During the 1999 national census a filtering survey called "Everyday Life and Health" was added. A total of 2,275 geographical areas were picked at random from the 1990 survey, stratified by departments nested within regions, by family, and by socio-professional statistics. The survey consisted of a self-administered 18-item questionnaire that collected information on activities of daily living. Ultimately, 2,223 of the 2,275 geographical areas (97.7%) collaborated in the Everyday Life and Health survey. From the 399,784 questionnaires distributed, 359,010 were completed and returned (89.1%). Questionnaires were to be answered by (or for) all members of a household. This survey did not check the validity of proxy respondents. Non-French speakers having no translation support were unable to answer the questionnaire.

2. Subjects from the Everyday Life and Health survey were clustered into six impairment groups ranging from no impairment (group 1) to severe impairment (group 6), based upon an impairment severity score [27]. Subjects in the severe impairment group had a higher probability of being detected by the Handicap-Dependency survey than did those in the Everyday Life and Health survey [22]. This over-sampling method made it possible to describe the consequences of impairments in detail, since subjects with impairments were over-represented in the Handicap-Dependency survey. Face-to-face interviews were available for 16,945 (77.8%) of 21,760 subjects selected at random from the 'Everyday Life and Health' respondents.

### Experimental design: the institution survey

Institutions were selected at random from the French Health Ministry files; day-care centres were not included. The sample was stratified according to eighteen strata [24]. The probability of selecting an institution was inversely proportional to the number of institutions in its stratum and proportional to its number of beds. Eight subjects were picked at random by the interviewers from each resident list.

In 1998, 2,075 institutions were selected and 155 of them (7.5%) refused to participate. The three most frequent reasons for refusal were lack of time (22.7%), the non-compulsory character of the INSEE survey (10.7%), and lack of staff to help the interviewer (7.3%). In total, 14,611 interviews (94.9%) were performed with 15,403 randomly selected subjects. Analyses were performed on 14,603 subjects with documented impairments, except for eight cases where interviews were stopped before impairments could be documented.

### Data collected

The survey documented blindness and low vision as declared by subjects, with no medical input. Three formal questions specific to vision were asked during the interview: (1) "Do you have trouble reading newspapers, books, etc ... with spectacles, if you use them?" (2) "Do you have trouble recognising the features of someone standing four meters away from you (with spectacles or contact lenses, if you usually use them)?" (3) "Would you say you are completely blind (light perception at the best), partially blind (still-form perception), or visually impaired?". Data were collected descriptively and experts in medical coding performed *post hoc *classifications of declared diseases. Thus, subjects were classified as belonging to one of the following groups: (1) blind; (2) low visual acuity; or (3) control (i.e. neither blind nor low vision). The cause of impairment was elicited by an open-ended question: "What is the cause of the stated impairment?" The free text was then coded by the interviewer under one of four broad categories: disease, birth-related, injuries, others.

Ophthalmologists' demography was derived from national statistics [21,28] published by the French Ministry of Health (*Ministère de la Santé et des Solidarités*). The *Direction de la Recherche, des Études, de l'Évaluation et des Statistiques *is in charge of up-dating the ophthalmologists' demography, amongst others statistics. We used 2002 data as proxy for regional eye-care services.

### Statistical analysis

Analyses were conducted with SAS Institute (North Carolina) software release 8.2. Weights for extrapolating data to the entire population were estimated by INSEE from the 1999 national census. These weights were applied to the Everyday Life and Health survey of impairment severity, refusal to participate in the Handicap-Dependency survey, and age, gender, size of household, type of household and geographical area size based on the latter survey. For the institution survey, weights included size of strata, the institution occupation rate (number of subjects in the institution/number of available beds), and the answer refusal rate (higher in psychiatric centres).

A weighted logistic regression was used to identify risk factors. One regression identified the risk factors for blindness (blind *versus *no visual impairment) and another the risk factors for low vision (low vision *versus *no visual impairment). The reference state was "no visual impairment". Risk factors included in the models were age (continuous variable), job classification (Reference farmer) and national region (Reference *Ile de France*). Odds-ratios with 95% confidence limits are presented. Age and job classification factors were chosen to adjust on the socio-economic variability amongst French regions (e.g., people living on the French Riviera are older and richer than those living in the North).

## Results

Altogether, 16,945 questionnaires were collected by the community survey and 14,603 by the institution survey. 2,703 subjects declared low vision and 350 blindness. Extrapolation to the national level predicted that 664,253 of 58,096,060 subjects (1.14%) lived in institutions. People living in institutions were older and less frequently male than those in the community. Additional descriptive information (socio-demographics, co-morbidity, etc ...) of this population can be found elsewhere [22-24].

Prevalences of low vision and blindness increased exponentially with age (Table 1). More than one-quarter of patients older than 90 years declared a visual impairment. Responses of centenarians were few and should be interpreted with caution.

**Table 1:** Prevalence of low vision in all populations (persons living at home or in institutions). 95% confidence interval. n.e. not estimable

**Age (years) **Persons living in institution and at home (n = 58,096,060)	**Prevalence of low vision**	**Prevalence of blindness**
0–9	0.62% [0.32%,1.20%]	<0.001% [0.00%,2.37%]
10–19	0.27% [0.12%,0.60%]	0.03% [0.00%,0.45%]
20–29	1.34% [0.77%,2.33%]	0.02% [0.00%,0.42%]
30–39	0.29% [0.14%,0.63%]	0.02% [0.00%,0.39%]
40–49	1.91% [1.13%,3.21%]	0.06% [0.01%,0.59%]
50–59	1.30% [0.73%,2.31%]	0.11% [0.01%,0.99%]
60–69	3.06% [1.82%,5.11%]	0.21% [0.03%,1.65%]
70–79	5.92% [3.64%,9.48%]	0.09% [0.01%,0.97%]
80–89	14.10% [8.90%,21.62%]	0.91% [0.12%,6.4%]
90–99	23.13% [14.18%,35.41%]	4.73% [0.68%,26.43%]
100 +	33.71% [n.e.]	3.27% [n.e.]

The major cause of blindness declared by subjects was acquired diseases (Table 2). 21,600 blindness were congenital and 35,000 were acquired. The figures for LV were 179,000 and 660,000, respectively. About 160,800 instances of blindness and low vision could be avoided in France by accident prevention.

**Table 2:** Causes of blindness and low vision declared by the respondents.

**Cause declared by respondent**	**Low vision**	**Blind**
Pregnancy and/or birth complications, congenital or hereditary disease	14.9%	30.9%
Acquired diseases	55.3%	50.2%
Injuries	12.4%	12.0%
Other or unknown	17.4%	6.9%

The prevalence of visual impairment was similar between the sexes. After adjustment on age and region, people exempted from employment, and those working as artisans, shopkeepers or business-owners, had significantly less risk of developing low vision (odds-ratios 2.10 and 1.51, respectively) than did farmers (Table 3). Persons with no professional activity had a higher probability (odds-ratio 0.28) of developing blindness than farmers.

**Table 3:** Probability of developing low vision or blindness according to job classification, adjusted on age and region. An OR greater than 1 means less risk of visual impairment. Reference category is 'Farmer'.

	**Low vision**		**Blindness**	
	**OR**	**95% CL**	**OR**	**95% CL**
Farmer (Reference)	1.00	na	1.00	na
Artisan, shopkeeper, business owner	1.51	1.12–2.03	0.91	0.33–2.51
Exempt	2.10	1.34–3.28	1.40	0.29–6.71
Part-time worker	1.08	0.79–1.47	3.60	0.42–31.09
Employee	0.96	0.71–1.32	0.91	0.27–3.03
Unskilled worker	0.97	0.77–1.21	1.38	0.53–3.62
No professional activity	1.11	0.66–1.86	0.28	0.08–0.96
Unclassified	1.34	0.81–2.20	1.05	0.18–6.29

After adjustment on age (more old people live in the South of France) and job classification (GDP in northern regions is lower), the prevalence of blindness did not differ significantly (95% CL) between regions (Table 4). The picture was different for low vision. In seven regions the probability of developing low vision (odds-ratios between 0.35 and 0.59) was significantly higher than in Ile-de-France, and only one region had a statistically significant lower probability (odds-ratio 2.10). On comparing range extremes, people of the same age and job classification had 6.0 more risk of low vision if they lived in Poitou-Charentes than in Haute-Normandie.

**Table 4:** Probability of developing low vision or blindness according to region, adjusted on age and job categories. An OR greater than 1 means less risk of develop low vision. Reference category is "Ile de France", i.e. Paris and its suburb.

	**Low vision**		**Blindness**	
	**OR**	**95% CL**	**OR**	**95% CL**
Ile de France (Reference)	1	na	1	Na
Champagne Ardennes	0.82	0.48–1.43	2.38	0.10–58.00
Picardie	0.59	0.37–0.96	0.90	0.11–7.15
Haute-Normandie	0.35	0.23–0.53	1.27	0.13–12.01
Centre	0.52	0.35–0.77	1.36	0.22–8.55
Basse-Normandie	1.57	0.72–3.44	2.79	0.09–88.38
Bourgogne	0.41	0.27–0.62	2.17	0.11–43.46
Nord-Pas-de-Calais	0.63	0.43–0.91	1.34	0.24–7.52
Lorraine	0.73	0.48–1.13	1.82	0.20–16.67
Alsace	1.62	0.88–2.97	1.35	0.16–11.11
France-Comté	1.95	0.86–4.45	0.87	0.10–7.45
Pays de Loire	0.45	0.31–0.64	1.65	0.24–11.38
Bretagne	1.38	0.85–2.26	1.16	0.23–5.75
Poitou-Charentes	2.10	1.04–4.23	1.39	0.19–10.34
Aquitaine	0.49	0.34–0.73	0.76	0.19–3.10
Midy-Pyrénées	1.20	0.70–2.07	1.47	0.21–10.37
Limousin	0.47	0.27–0.81	3.88	0.03–442.19
Rhônes-Alpes	0.74	0.52–1.06	0.87	0.23–3.22
Auvergne	0.62	0.36–1.04	2.24	0.08–59.30
Languedoc-Roussillon	0.75	0.47–1.20	0.64	0.15–2.77
Provence-Alpes-Côtes d'Azur	0.77	0.54–1.12	0.64	0.20–2.03

**Figure 1:**
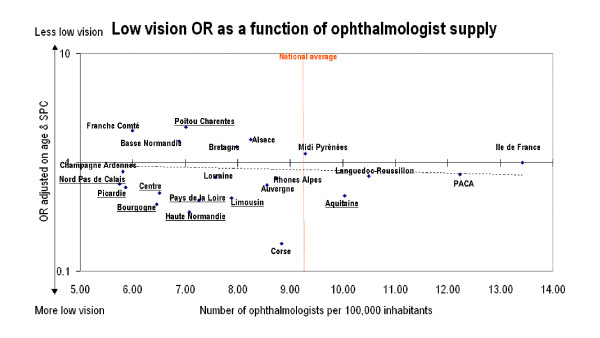
Relationship between the number of ophthalmologists per 100,000 inhabitants and the OR (adjusted on age and socio-professional categories) for low vision per region. Reference: "Ile de France". Dotted line: linear regression. Region ORs that differed significantly from 1 are underlined. An OR greater than 1 means less risk of developing low vision. PACA: Provence-Alpes-Côtes d'Azur.

A [non-significant] linear trend in Figure 1 indicates that the probability of low vision decreases as the density of ophthalmologists (number of ophthalmologists per 100,000 inhabitants) increases, after adjustment on age and job classification. Also, six of the seven regions with significantly higher prevalence rates of low vision had ophthalmologist densities below the national average.

## Discussion

The surveys analysed shared two limitations: (1) their cross-sectional design did not allow an analysis of possible causalities between blindness, or low vision, and risk factors; and (2) the actual visual acuity of subjects who responded was not measured by ophthalmologists. Subjects classified as blind self-declared that they could not perceive shapes. This may be a serious limitation to our analyses, although our prevalence figures are close to the only French report in the international literature [29]. On the other hand, we did study representative samples of subjects from both the community and institutions. Another issue concerns the small number of subjects who declared themselves blind, which resulted in large OR confidence intervals.

The different relationships between age and the prevalence rates of low vision and blindness may be explained by the different reasons given by subjects for the impairments. A considerable proportion of blindness was related to pregnancy and childbirth, whereas the main cause of low vision was attributed to acquired diseases. In other words, a significant proportion of blindness is not managed by ophthalmologists, which might explain the lack of association between ophthalmologist density and the blindness prevalence rate Lastly, most diseases affecting vision in developed countries do not make patients immediately blind since treatments are available and costs reimbursed. Therefore, most patients have had low vision before becoming blind. However, since the cause of visual impairment was self-declared and was not medically certified, apparent differences between the causes of low vision and blindness might be explained by recall bias.

It should be noted that one-in-eight visual impairments were related to injury. Therefore, preventative measures would have avoided some cases of low vision and blindness, which totalled 152,400 and 8,400 total persons, respectively, for a country with 58,000,000 inhabitants.

Persons with higher educational achievement were less at risk for low vision, but this was not so for blindness. Higher education enables people to become better informed about potential diseases related to ageing, and gives them more effective access to healthcare.

When the present data were collected, access to ophthalmologists in France did not require referral by general practitioners. In addition, more than 95% of French people have private insurance supplementing their national sick fund protection [30]. Insurance policies cover hospitalisation costs and all out-patient care: drugs, visits, examinations, etc ... Average patient co-payment in 2001 was 11.1% of total expenditure [31]. These financial provisions were supposed to ensure excellent equity. What we found, however, was inequity.

It could be expected that people with greater economic means or greater educational levels might be much more aggressive in seeking out eye care and some of them might even be seeking eye cares outside their area. This is why it was very important to get prevalence rates adjusted on job description to control for the above effect. In France, most of the vision is under the control of ophthalmologists: visual acuity, diagnosis, treatments, etc ... There is no limitation to access them, outside their availability. The role of optometrists is very low, almost inexistent. Therefore, the ophthalmologist density could be considered as a good indicator of resources available to preserve vision at a national level.

After adjusting on age and job classification, our analysis showed that differences existed between geographic regions with respect to the prevalence of low vision. Subjects living in Haute-Normandie had a 2.86 greater chance of developing low vision than people in the Paris area, whereas persons in Poitou-Charentes had a 2.10 lower chance than Parisians. In contrast, an association was found between ophthalmologist density (number/100,000 inhabitants) and the regional distribution of low vision. Thus, seven of eight regions (85.7%) with a significantly higher prevalence of low vision had ophthalmologist densities below the national average. This suggests that the supply of vision-related services may be a determinant of eye morbidity at a national level.

To confirm these findings, it would be worthwhile to study the relationship between regional visual impairment rates and indicators of other eye-care activities, such as number of visits/inhabitant, glaucoma diagnosis campaign, etc.. Inequality of quality of care could also be a factor explaining the prevalence rate differences across the regions. Unfortunately, such aspects of ophthalmological activity or quality estimates are unavailable in France at a regional level Lastly, the same HID surveys showed that visual impairment impacted dramatically on activities of daily living [22-24] and had economic consequences on the family revenue [7]. The latter, alone, might reduce access to eye-care. Hence, to conclude that a similar density of ophthalmologists should be provided in all regions is premature.

However, the causality of the association between the prevalence of visual impairment in relation to the number of ophthalmologists in a given area might be confounded by some factors that were not collected in our surveys. This encompasses, for example, population genetic factors distribution across the different areas, other health care resource supply (access to hospital is more difficult in rural area), or eating habits (south part of France people used to eat more fresh fruits and vegetables which is known to protect against acquired visual impairment). These are strong limitations to the analyses we conducted and additional data should be collected to confirm our findings.

It is interesting that a recent national survey of the UK system for delivering care to low vision subjects, involving a wide range of service providers, also found regional inequity, as in France [32]. The number of service providers was lowest in areas where the general population was small, but the prevalence of low vision was highest. Conversely, the number of service providers was highest in cities where the general population was large, despite the prevalence of low vision being only moderate.

It is evident that where practitioner remuneration is based on a fee-for-service, as in France, measures are needed to control physician-induced demand. However, on a broader scale, irrespective of the healthcare system, there is some evidences to justify including a minimum level of equity in plans to reorganise eye-care services. For example, the prevalence of visual impairment in the Auvergne does not differ significantly from the Ile-de France, yet the density of ophthalmologists is below the national average. It would be equitable if such standard were applied to all regions.

It was not the intention of this paper to demonstrate or claim the need for a fixed ratio of ophthalmologists to inhabitants. However, investment in healthcare is supposed to be effective, as resources are limited. Ultimately, the daily work of ophthalmologists is to preserve vision, so maintenance of vision or reduction of low vision prevalence rates is a legitimate public health aim. We found some weak associations. This suggests that a minimum ophthalmologist density might be an aspect to consider when allocating resources for the preservation of vision.

## Conclusion

An association was found between the number of ophthalmologists/inhabitants and the prevalence of low vision, in France. These data suggests that ophthalmologist density could be one of the drivers of good vision at a population level.

## Competing interests

The author(s) declare that they have no competing interests.

## Authors' contributions

AL and FF retrieved the data bases. The analyses were performed GB. All authors contributed to the writing of the manuscript.
